# Dr. William Thompson Lusk

**Published:** 1897-07

**Authors:** 


### Obituary.

Dr. William Thompson Lusk, president of the Bellevue Hospital Medical College and professor of obstetrics and gynecology in that institution, died suddenly at his home, No. 47 East Thirty-fourth street, in the city of New York, Saturday afternoon, June 12, 1897, of cerebral apoplexy. He was apparently in his usual health in the morning and the suddenness of his death shocked a whole community.

Dr. Lusk was born at Norwich, Conn., in 1838. His father was a descendant of an old Norwich family. William T. Lusk was in the class of '59 at Yale, but left the college after a year. In 1864 he was graduated from the Bellevue Hospital Medical College. Soon after he had received his degree he went to Europe, and continued the study of medicine at Heidelberg, Berlin, Edinburgh, Paris, Vienna and Prague. On returning to this country in 1868 he became professor of physiology in the Long Island College, where he remained until 1871. In 1870 and the following year he gave lectures on physiology at Harvard.

Dr. Lusk was also the visiting physician of the Nursery and Children's Hospital in 1870 and 1871, and visiting physician to the Charity Hospital in the same years, consulting physician to the Maternity Hospital, to the New York Foundling Asylum and to the New York Lying-In Hospital. He was also the visiting gynecologist to St. Vincent's Hospital. In 1871 he was editor of the New York *Medical Journal*. In 1875 he was the vice-president of the New York Obstetrical Society, and later its president. He has also been president of the American Gynecological Society.

At the beginning of the Civil War, Dr. Lusk joined a New York volunteer regiment as a private soldier. For meritorious performance in the discharge of his duties he was made second lieutenant, and later was promoted to be captain and assistant adjutant-general. He was a companion of the Military Order of the Loyal Legion.

In 1872 he received the honorary degree of A. M. from Yale, and later the honorary degree of LL.D. from the same institution.

His principal work was the Science and Art of Midwifery, which has been translated into many languages. He was also the author of the following monographs: Histological doctrines of M. Robin; Uremia a common cause of death in uterine cancer; Irregular uterine action during labor; Inquiry into the pathology of uterine cancer; Clinical report of the lying-in service at Bellevue Hospital for 1873; Origin of diabetes, with some new experiments regarding the glycogenic function of the liver; Cephalotribes and Cephalotripsy; Genesis of an epidemic of puerperal fever; Morphia in childbirth; Nature, causes and prevention of puerperal fever. These and many others bespeak his literary accomplishments.

Dr. Lusk was a forceful teacher, with few equals and no superior at least in the United States; and he had achieved a renown in his special field of study that extended to all quarters of the globe. Personally he was one of the most delightful of men, possessed of an attractive, clear, musical voice, and a modest, charming, fascination of manner that gave him a strong hold upon the social, professional and public sides of life. He died at the zenith of a fame that was deservedly won by one of the most gifted members of the medical profession that has risen from obscurity to renown in the last half of the nineteenth century.

Dr. Lusk was married twice and leaves two sons and three daughters. The Hon. S. V. Chittenden, of Brooklyn, father of his first wife, erected to her memory a beautiful building at Yale University. His eldest son, Graham Lusk, is professor of physiology at Yale University, while the younger, Dr. Wm. Chittenden Lusk, is chief of the surgical clinic at Bellevue Hospital Medical College.

